# Nebulized Long-Acting Bronchodilators to Treat Acute Respiratory Failure in an Older Adult: A Case Report

**DOI:** 10.7759/cureus.80218

**Published:** 2025-03-07

**Authors:** Avery North, Katelyn Helwig, Macie Gibbs, Jordan Fuller, Paul M Boylan

**Affiliations:** 1 Medicine, The University of Oklahoma Health Sciences Center College of Pharmacy, Oklahoma City, USA; 2 Clinical and Administrative Sciences, The University of Oklahoma Health Sciences Center College of Pharmacy, Oklahoma City, USA

**Keywords:** acute respiratory distress syndrome (ards), albuterol, arformoterol, nebulization, revefenacin

## Abstract

This report describes the case of a 79-year-old man admitted to the intensive care unit (ICU) for acute hypoxemic respiratory failure due to coronavirus disease 2019 (COVID-19) and treated with long-acting nebulized bronchodilators, arformoterol and revefenacin. This article aimed to assess the use of long-acting nebulized bronchodilators in an older adult with acute respiratory failure. The institutional setting was a 500-bed academic medical center in the southern US. Arformoterol is a long-acting beta agonist (LABA) and revefenacin is a long-acting muscarinic antagonist (LAMA), both approved to treat stable chronic obstructive pulmonary disease (COPD). Neither medication is US Food and Drug Administration approved for acute respiratory failure. In this case, both medications were used for their rapid-onset and long-duration bronchodilator activity to treat acute respiratory failure secondary to COVID-19. This older adult clinically improved within three days of nebulized long-acting bronchodilator use.

## Introduction

Acute respiratory distress syndrome (ARDS) is a form of acute respiratory failure characterized by hypoxemia and pulmonary edema secondary to direct lung injuries (e.g., pneumonia, aspiration, or toxic inhalants) or indirect lung injuries (e.g., trauma or sepsis) [1]. Severe coronavirus disease 2019 (COVID-19) pneumonia may lead to ARDS [2]. Mortality and morbidity attributed to COVID-19 is largely due to viral pneumonia-induced ARDS, which disproportionately develops in elderly patients [2]. Advanced age is a known risk factor for developing severe respiratory infections and complications, including COVID-19 and ARDS, and these patients often require intensive care unit (ICU) admission and extensive therapy for management [2,3].

The current recommendations for management of ARDS, including cases caused by COVID-19 pneumonia, include mechanical ventilation, prone positioning, systemic corticosteroids, venovenous extracorporeal membrane oxygenation, and neuromuscular blocking agents, though the guidelines grade several of these recommendations as conditional given low-certainty evidence [4,5]. Inhaled bronchodilators are not included in these guidelines, and their place in critical care is unclear. A 2019 Cochrane systematic review and meta-analysis did not identify statistical differences or clinical improvements in mortality or ventilator-free days among patients treated with the short-acting beta agonist, albuterol [6]. Pharmacologically, short-acting beta agonists may worsen ARDS by increasing heart rate and pulmonary fluid clearance and thus increase myocardial demand and cause lung hyperinflation, respectively [7,8].

Inhaled bronchodilators, notably long-acting agents, have a proven benefit in chronic obstructive pulmonary disease (COPD) exacerbations [9]. Long-acting bronchodilators provide additional benefits in the treatment of these exacerbations, as they have comparable efficacy and safety to short-acting bronchodilators with fewer administrations required per day [10,11]. The long-acting beta agonist, formoterol, is also connoted as a fast-acting bronchodilator because it has a similar rapid onset of action like the short-acting beta agonist, albuterol, but possesses the added benefit of extended (12 hour) bronchodilatory activity [7]. Revefenacin is the first nebulized long-acting muscarinic antagonist approved for once-daily use in patients with moderate to severe COPD [12]. When used as maintenance treatment, it demonstrated significant improvements in pulmonary function (i.e., trough forced expiratory volume in 1 second (FEV1) increased by 100 mL) and was nominally similar to tiotropium with respect to the incidence of acute exacerbations of COPD (revefenacin 21.8% vs tiotropium 28.1%, p-value unreported). Overall, revefenacin is well tolerated, with a low incidence of systemic antimuscarinic adverse events (e.g., xerostomia, headache). Revefenacin has not been studied in the setting of acute respiratory failure, but it may be a reasonable choice for patients in the ICU due to its nebulized route of administration, once-daily dosing, and rapid onset of bronchodilator activity.

This case report aimed to describe and discuss the use of long-acting nebulized bronchodilators in an older adult with acute respiratory failure secondary to COVID-19. This case was approved by the affiliated institutional review board (protocol #16690, approved January 25, 2024, Category 5) and prepared by the CARE guidelines for case reporting [13]. This case was previously presented as a poster at the 2023 American Society of Health-System Pharmacists Annual Meeting on December 5, 2023.

## Case presentation

A 79-year-old man presented to the emergency department of a 500-bed academic medical center in the southern US as a transfer from a small, rural county hospital. He presented with tachypnea (respiratory rate 26 breaths per minute), hypoxia (oxygen saturation 89%), fever (39.3°C rectal), tachycardia (106 beats per minute), hypertension (126/99 mm Hg), and dyspnea occurring both at rest and with activity. Physical exam revealed rales and decreased bilateral breath sounds without accessory muscle use. His oxygen saturation increased to 92%, requiring 4 to 5 L via nasal cannula. Notable laboratory results on admission are presented in Table 1. The etiology of elevated troponins was considered multifactorial: COVID-19 may cause myocardial ischemia and thus increase troponin levels, and this patient living with chronic kidney disease requiring dialysis missed at least one dialysis session due to his acute illness [14]. Urinalysis was negative for infection.

**Table 1:** Laboratory values BG = blood gas; dL = deciliters; g = grams; L = liters; microL = microliters; mg = milligrams; mm Hg = millimeters of Mercury; mmol = millimoles; ng = nanograms

Measurement (unit)	Patient result	Reference range
Sodium (mmol/L)	137	137-146
Potassium (mmol/L)	4.1	3.5-5.2
Chloride (mmol/L)	95	98-111
Carbon dioxide (mmol/L)	24	19-29
Blood urea nitrogen (mg/dL)	40	8-22
Serum creatinine (mg/dL)	5.7	0.78-1.34
Glucose (mg/dL)	125	68-116
Calcium (mg/dL)	8.6	8.7-10.1
Aspartate aminotransferase (units/L)	100	19-46
Alanine aminotransferase (units/L)	31	12-47
Alkaline phosphatase (units/L)	80	35-129
White blood cells (million cells/microL)	6.8	4-11
Red blood cells (cells/microL)	3.7	4.5-5.9
Hemoglobin (g/dL)	11.5	13-18
Hematocrit (%)	35	39-52
Lactic acid (mmol/L)	4.7	0.5-1.9
C-reactive protein (mg/L)	49	<5
Troponin (ng/L)	1,470	<15
BG pH	7.46	7.38-7.41
BG pCO_2_ (mm Hg)	40	41-47
BG pO2 (mm Hg)	39	22-37
BG bicarbonate (mmol/L)	28	22-29

A chest x-ray revealed right-sided pneumonia and was negative for pleural effusion. The patient’s primary problem was identified as acute hypoxemic respiratory failure secondary to COVID-19, confirmed via polymerase chain reaction at the academic medical center. Intravenous cefepime 2 gram was initiated at the rural county hospital and continued (at a 1-gram dose, given the patient’s renal impairment) at the academic medical center given concerns for a superimposed bacterial pneumonia in addition to COVID-19. His past medical history included hypertension, benign prostatic hyperplasia, and end-stage renal disease requiring hemodialysis three days per week. On this hospital admission, he was diagnosed with new-onset atrial fibrillation. His home medications are listed in Table 2. Outpatient records confirmed the patient did not have a prescription for inhaled bronchodilators prior-to-admission, and he denied any previous diagnosis of COPD or asthma. He reported a cigarette smoking history and quit 59 years ago; he denied alcohol and drug use. Per the state immunization registry, the patient received one dose of the viral vector COVID-19 vaccine 16 months prior to this event. Isolation precautions, both airborne and droplet, were placed on admission and continued throughout the hospitalization.

**Table 2:** Prior-to-admission home medications

Medication	Directions
Calcium acetate 667 mg capsules	Take 2 capsules by mouth before meals three times daily
Clonidine 0.2 mg tablets	Take 1 tablet by mouth three times daily as needed for blood pressure above 160/110 mm Hg
Metoprolol succinate 100 mg tablets	Take 1 tablet by mouth every other day
Nifedipine extended release 60 mg tablets	Take 1 tablet by mouth every morning
Tamsulosin 0.4 mg capsules	Take 1 capsule by mouth every morning

Upon ICU admission, the patient required continuous bilevel positive airway pressure. He received one 200 mg intravenous dose of remdesivir and one 6 mg intravenous dose of dexamethasone for COVID-19. During his stay, both in the ICU and general medical unit, the patient received three nebulized, once-daily doses of revefenacin 175 mcg for acute hypoxemic respiratory failure. Doses 1 and 2 were administered on the last two days of ICU stay (days 2 and 3), and dose 3 was administered four days after ICU discharge. In addition to revefenacin, the patient also received arformoterol 15 mcg nebulized every 12 hours for treatment of his acute respiratory failure. Orders for an albuterol 90 mcg metered dose inhaler and albuterol-ipratropium 2.5/0.5 mg nebulizer (both with directions to use every six hours as needed for dyspnea) were placed through no doses were required or administered. During the evening of ICU day 2, the patient developed mental status changes and acute agitation, which was treated with 7.5 mg of intramuscular olanzapine; the interprofessional team felt the mental status changes were due to comorbid pneumonia and thus intravenous cefepime 1 gram intravenously daily was changed to ceftriaxone 2 grams intravenously daily. Nonetheless, the Naranjo nomogram for estimating the probability of adverse drug events was completed (for revefenacin) and is depicted in Table 3 [15].

**Table 3:** Naranjo nomogram to estimate the probability of adverse drug reaction – revefenacin and agitation Adapted from [15]. Permission obtained from Springer Nature (January 31, 2025).

Question	Answer and score
Are there previous conclusive reports on this reaction?	No, 0 points
Did the adverse event appear after the suspected drug was administered?	Yes, 2 points
Did the adverse reaction improve when the drug was discontinued or a specific antagonist was administered?	No, 0 points
Did the adverse reaction reappear when the drug was readministered?	No, -1 point
Are there alternative causes that could have on their own caused the reaction?	Yes, -1 point
Was the drug detected in the blood in concentrations known to be toxic?	Unknown, 0 points
Was the reaction more severe when the dose was increased, or less severe when the dose was decreased?	Unknown, 0 points
Did the patient have a similar reaction to the same or similar drugs in any previous exposure?	No, 0 points
Was the adverse event confirmed by any objective evidence?	No, 0 points
Total score and probability	0, unlikely or doubtful

After three days, the patient was discharged from the ICU and resolution of respiratory failure was noted on the patient chart. His total ICU length of stay was four days and hospital length of stay was 16 days (for the treatment of COVID-19 pneumonia and atrial fibrillation with rapid ventricular response).

## Discussion

Historically, long-acting bronchodilators may have been avoided to treat acute respiratory conditions because of Black Box Warnings for increased asthma-related mortality [16]. Although long-acting bronchodilators are not included in the guideline-recommended management of respiratory failure, there are plausible pharmacologic benefits warranting their exploration in such cases. When compared to short-acting bronchodilators, novel long-acting bronchodilators (i.e., revefenacin, arformoterol) were shown to have comparable safety and efficacy when used to treat acute exacerbations of COPD (ECOPD) [10,11]. Although revefenacin has a long-acting duration, it has a short onset of action (14 to 41 minutes), thereby providing relief quickly and maintaining bronchodilation over 24 hours [12]. By providing sustained bronchodilation, it may decrease the number of rescue bronchodilator administrations required during hospital stays, such as in this case wherein the patient did not require either albuterol metered dose inhalers or albuterol-ipratropium nebulizers. At our institution, metered dose inhalers and nebulized medications must be administered by nurses and respiratory therapists, respectively. Administration and monitoring times for these short-acting rescue medications may require 15 minutes of direct patient care observation. Thus, administration of nebulized long-acting bronchodilators may reduce the burden of rescue medication on both patients and healthcare staff [17].

The safety of long-acting bronchodilators is similar to short-acting bronchodilators [9]. However, systemic exposure of revefenacin is increased in patients with renal and hepatic impairment, therefore these patients should be monitored more closely for anticholinergic adverse effects, particularly in patients at risk for agitation, especially older adults [12]. Though it is doubtful that revefenacin alone or in combination with concomitant risk factors caused this patient’s ICU delirium, consideration may be warranted. The 2023 American Geriatrics Society Beers Criteria for potentially inappropriate medication use in older adults provides a strong recommendation against the use of medications with anticholinergic properties (i.e., antihistamines and antidepressants with strong anticholinergic properties) be avoided because of increased risks for confusion, dementia, and sedation [18]. Safety signals have not been identified when long-acting bronchodilators were used in a sample of 30 adults admitted with acute ECOPD [10]. Further observational studies are necessary to corroborate these findings and affirm the safety of long-acting bronchodilators in both intensive care and general medicine settings.

This case report possesses limitations. This patient received prompt treatment with remdesivir and dexamethasone for COVID-19, which likely contributed to this patient’s overall improvements and brief ICU length of stay. Additionally, there was a four-day lapse between doses 2 and 3 of the revefenacin for reasons undocumented in the medical record or discussed during interprofessional patient care rounds. Despite the prompt resolution of this patient’s COVID-19 pneumonia, nebulizer utilization during COVID-19 is controversial because of the risk for aerosolizing the virus and increasing transmission to healthcare providers, caregivers, and contacts [9]. Contact precautions, such as those implemented in this case report, should be strictly adhered to if nebulized bronchodilators are administered to patients with airborne illnesses.

## Conclusions

Although long-acting bronchodilators are not approved to treat acute respiratory failure, the older adult in this case clinically improved after three doses of revefenacin and arformoterol. Additional studies are necessary to determine the efficacy and safety of long-acting bronchodilators to treat acute respiratory distress syndrome or acute exacerbations of chronic lung conditions. Further analysis on the incidence of adverse events, particularly for patients in the ICU, is also warranted.
